# Circulating Plasma Gelsolin: A Predictor of Favorable Clinical Outcomes in Head and Neck Cancer and Sensitive Biomarker for Early Disease Diagnosis Combined with Soluble Fas Ligand

**DOI:** 10.3390/cancers12061569

**Published:** 2020-06-13

**Authors:** Chen-Tzu Chiu, Pei-Wen Wang, Meshach Asare-Werehene, Benjamin K. Tsang, Dar-Bin Shieh

**Affiliations:** 1 Institute of Basic Medical Sciences, National Cheng Kung University, Tainan 70101, Taiwan; dinosaurchiu@gmail.com; 2 Institute of Oral Medicine and Department of Stomatology, College of Medicine, National Cheng Kung University Hospital, National Cheng Kung University, Tainan 70101, Taiwan; peiwen1005@gmail.com; 3 Center of Applied Nanomedicine, National Cheng Kung University, Tainan 70101, Taiwan; 4 Departments of Obstetrics & Gynecology and Cellular & Molecular Medicine, University of Ottawa, Ottawa, ON K1H 8L6, Canada; masarewerehene@yahoo.com (M.A.-W.); btsang@ohri.ca (B.K.T.); 5 Chronic Disease Program, Ottawa Hospital Research Institute, Ottawa, ON K1H 8L6, Canada; 6 Center for Micro/Nano Science and Technology, National Cheng Kung University, Tainan 70101, Taiwan

**Keywords:** plasma gelsolin, sFasL, circulating biomarker, head and neck cancer, diagnosis

## Abstract

Head and neck cancer (HNC) accounts for more than 330,000 cancer deaths annually worldwide. Despite late diagnosis being a major factor contributing to HNC mortality, no satisfactory biomarkers exist for early disease detection. Cytoplasmic gelsolin (cGSN) was discovered to predict disease progression in HNC and other malignancies, and circulating plasma gelsolin (pGSN) levels are significantly correlated with infectious and inflammatory disease prognoses. Here, the plasma levels of five candidate biomarkers (circulating pGSN, squamous cell carcinoma antigen, cytokeratin 19 fragment, soluble Fas, and soluble Fas ligand (sFasL)) in 202 patients with HNC and 45 healthy controls were measured using enzyme-linked immunosorbent assay or Millipore cancer multiplex assay. The results demonstrated that circulating pGSN levels were significantly lower in patients with HNC than in healthy controls. Moreover, circulating pGSN outperformed other candidate biomarkers as an independent diagnostic biomarker of HNC in both sensitivity (82.7%) and specificity (95.6%). Receiver operating characteristic curves indicated that combined pGSN and sFasL levels further augmented this sensitivity (90.6%) for early disease detection. Moreover, higher pGSN levels predicted improved prognosis at both 5-year overall survival and progression-free survival. In conclusion, circulating pGSN could be an independent predictor of favorable clinical outcomes and a novel biomarker for the early HNC detection in combination with sFasL.

## 1. Introduction

Head and neck cancer (HNC) accounts for more than 650,000 new cases and 330,000 cancer deaths annually worldwide [1], and it is the sixth most common cancer. In Taiwan, HNC is one of the most rapidly growing types of cancer among young (25–45-year-old) men. Moreover, the 5-year overall survival (OS) rate is only 59.8% for oral cancer and only 29.2% for hypopharyngeal cancer [2]. Precision medicine is an emerging trend in advanced disease management, including for cancers, where biomarkers that predict patients’ clinical outcomes and therapeutic responses play significant roles. For example, clinical trials have reported certain tyrosine kinase inhibitors such as gefitinib and erlotinib to be superior to traditional chemotherapy, but only in patients with lung cancer of corresponding EGFR mutations, particularly exon 19 deletion and L858R point mutation [3,4].

Biomarkers are particularly crucial in early disease stages because early intervention not only significantly enhances clinical outcomes and quality of life of the patients but also saves medical costs over the treatment course. Such precision biomarkers may also contribute to evidence-based optimization of treatment strategies by providing an understanding of the molecular signaling networks of the tumor. However, although many serum proteins, such as squamous cell carcinoma antigen (SCC-Ag), MMP9, and P16, have been proposed as useful circulating protein biomarkers for various cancers, none have been consistently reported for HNC. Nevertheless, other circulating biomarker types for HNC have emerged; these include circulating tumor cells, exosomal microRNA, and circulating tumor DNA [5,6,7]. Detection of these novel biomarkers require further advancement and approval in technology platforms. SCC-Ag in the circulation may suggest the presence of various human carcinomas such as oral, lung, and cervical cancers [8,9,10]. However, Yuan et al. found the sensitivity and specificity of SCC-Ag for HNC prediction to be only 73.37% and 68.1%, respectively [11]. Hence, the identification of novel biomarkers with superior test accuracy is crucial for detecting patients with HNC and guiding clinical therapeutics.

Defects in apoptosis regulation have been implicated in numerous human diseases, including cancers [12]. A critical apoptosis signaling pathway involves Fas/Fas ligand (FasL)-mediated signaling [13]. FasL expression in human HNC possibly potentiates an immunosuppressive function by promoting T lymphocyte apoptosis [14]. Gelsolin (GSN), an actin cytoskeleton-modulatory protein, can regulate not only cell morphology and motility but also cell apoptosis. We previously reported that cytoplasmic GSN (cGSN) expression in patients with HNC is strongly associated with their chemoresistant phenotype. cGSN overexpression also induces chemoresistance by inhibiting apoptosis initiated by chemotherapeutic agents [15,16]. Besides cytoplasmic form GSN, the same gene on chromosome 9 also encodes a secretory form, the plasma GSN (pGSN), through alternative splicing. pGSN is mainly secreted from skeletal and cardiac muscles [17] and is one of the most abundant plasma proteins in the circulation of vertebrates [18]. pGSN expression is implicated in various pathological conditions, such as acute respiratory distress syndrome, sepsis, acute hepatic failure, myonecrosis [19,20,21], and various cancers [22,23]. Recently, the role of pGSN in apoptosis induction in tumor-infiltrating CD8+ T lymphocytes has been demonstrated through FasL binding in prostate cancer [24]. However, the significance of circulating pGSN levels in HNC prognosis and the relevant clinicopathological implications warrant elucidation. In this study, the roles of pGSN as a biomarker for HNC and as the predictor of patients’ clinical outcome—either alone or in combination with other biomarkers—were evaluated.

## 2. Results

### 2.1. Patient Characteristics

The clinicopathologic characteristics of our patients with HNC are presented in Supplementary Table S1. Patient age ranged from 22 to 81 years (median, 53 years). Of the patients, 33 (16.3%), 52 (25.7%), 40 (19.8%), and 77 (38.1%) had TNM (tumor, node, metastasis) stage I, II, III, and IV disease, respectively. In terms of tumor differentiation, 104 (51.5%), 73 (36.1%), and 17 (8.4%) tumors were well, moderately, and poorly differentiated, respectively. The HNC tumor sites are listed in Supplementary Table S2. The median follow-up duration for all patients was 19.6 months (range, 1.0–202.0 months). During follow-up, 55 patients (27.2%) developed progressive diseases and 76 patients (37.6%) died. pGSN levels were measured, and the patients were dichotomized according to their median pGSN level (73.5 μg/mL) into low and high groups. 

### 2.2. Diagnostic Value of Circulating pGSN in Patients with HNC

**Figure 1:**
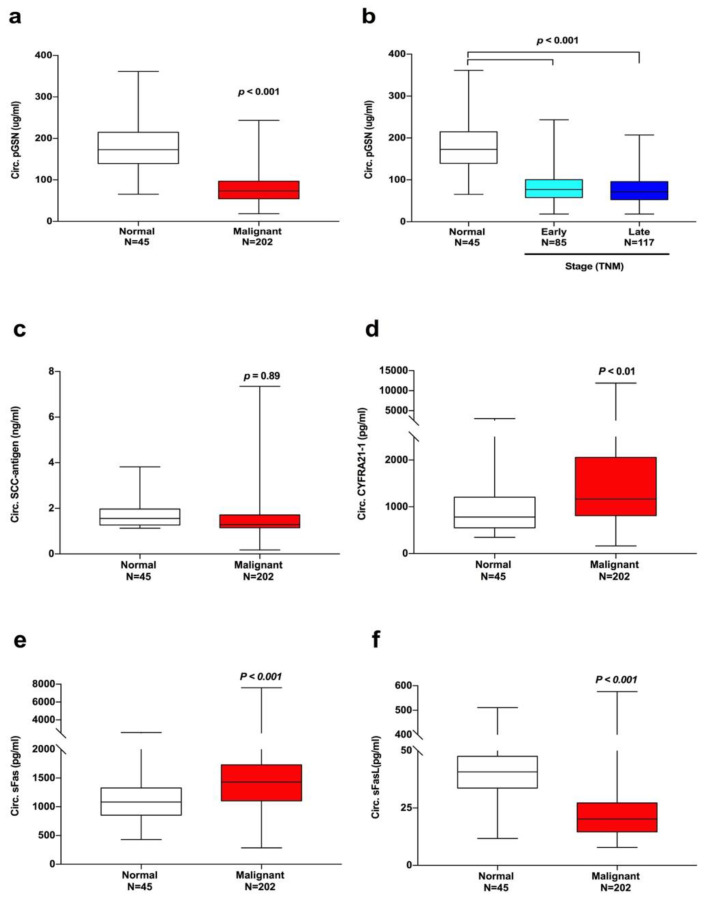
Circulating plasma gelsolin (pGSN) is the optimal diagnostic biomarker for head and neck cancer (HNC). (**a**) Circulating pGSN levels were significantly lower in patients with HNC (red bar, *n* = 202; 81.03 ± 38.14 μg/mL) than in healthy controls (white bar, *n* = 45; 181.7 ± 58.54 μg/mL; *p* < 0.001). (**b**) No significant difference existed between circulating pGSN levels in patients at early (stages I + II) versus advanced (stages III + IV) HNC stages, whereas the healthy controls presented distinctively higher circulating pGSN levels (*p* = 0.89). Circulating (**c**) squamous cell carcinoma (SCC) levels exhibited no significant differences between normal and malignant disease. Circulating (**d**) CYFRA21-1 and (**e**) soluble Fas (sFas) levels were significantly higher in patients with HNC than in healthy controls. (**f**) Circulating soluble FasL (sFasL) levels were significantly lower in patients with HNC than in healthy controls. Data are shown as mean ± SEM.

Enzyme-linked immunosorbent assay (ELISA) results revealed significantly lower circulating pGSN levels in patients with HNC (81.03 ± 38.14 μg/mL) than in the healthy controls (181.7 ± 58.54 μg/mL, *p* < 0.001; Figure 1a). Moreover, such predictive value was evident in patients with early-stage HNC (Figure 1b). Plasma levels of reported candidate circulating tumor biomarkers SCC-Ag, cytokeratin 19 fragment (CYFRA21-1), soluble Fas (sFas), and soluble FasL (sFasL) were also analyzed and compared with those of circulating pGSN. Diagnostic values for circulating SCC-Ag for HNC was not evident (*p* = 0.89, Figure 1c). CYFRA21-1 levels were significantly higher in patients with HNC (1704 ± 109.3 pg/mL) than that of the healthy controls (927.9 ± 79.38 pg/mL, *p* < 0.01; Figure 1d). In carcinogenesis, apoptosis pathways play crucial roles through Fas and FasL activation [25]. We discovered that the mean circulating sFasL levels was significantly lower in patients with HNC (66.89 ± 12.87 pg/mL) than in healthy controls (29.3 ± 3.596 pg/mL, *p* < 0.001). However, the mean sFas levels demonstrated the opposite trend (1538 ± 54.36 pg/mL in HNC group vs. 1111 ± 57.76 pg/mL in the control group, *p* < 0.001, Figure 1e,f). 

**Figure 2:**
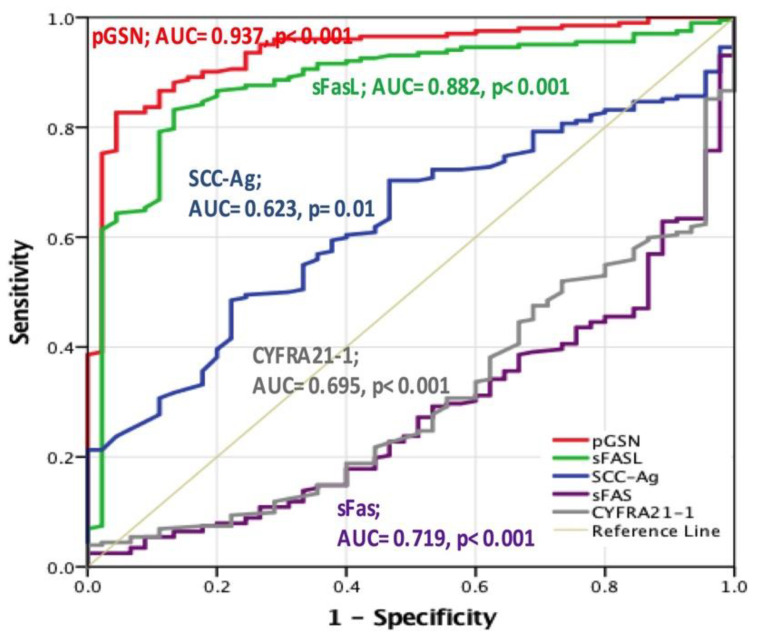
Receiver operating characteristic curves for all candidate circulating cancer biomarkers revealed pGSN to be the optimal predictor of HNC. The area under curve (AUC) of circulating pGSN was 0.937 (*p* < 0.001), whereas it was 0.882 for sFasL (*p* < 0.01), 0.695 for CYFRA21-1 (*p* < 0.001), 0.623 for SCC-Ag (*p* < 0.001), and 0.719 for sFas (*p* < 0.001).

The approximate AUC derived from the receiver operating characteristic (ROC) curve was used to assess the diagnostic performance of the candidate cancer biomarkers (Figure 2). The AUC of circulating pGSN was 0.937 (*p* < 0.001), whereas it was 0.882 for sFasL (*p* < 0.01), 0.695 for CYFRA21-1 (*p* < 0.001), 0.623 for SCC-Ag (*p* < 0.001), and 0.719 for sFas (*p* < 0.001). The optimal cutoff levels were set using Fisher’s exact test. It was 106.25 μg/mL for circulating pGSN, 30.15 pg/mL for circulating sFasL, 1401 pg/mL for sFas, 1.26 ng/mL for SCC-Ag, and 1568 pg/mL for CYFRA21-1. The sensitivity and specificity of pGSN were 82.7% and 95.6%, respectively. They were 83.2% and 86.7% for circulating sFasL; 53.0% and 86.7% for sFas, 48.5% and 77.8% for SCC-Ag, 37.6% and 95.6% for CYFRA21-1. It is thus concluded that circulating pGSN was the optimal predictive HNC biomarker, followed by sFasL and then by sFas.

### 2.3. pGSN as a Valuable Diagnostic Tool for Early HNC Stage

**Figure 3:**
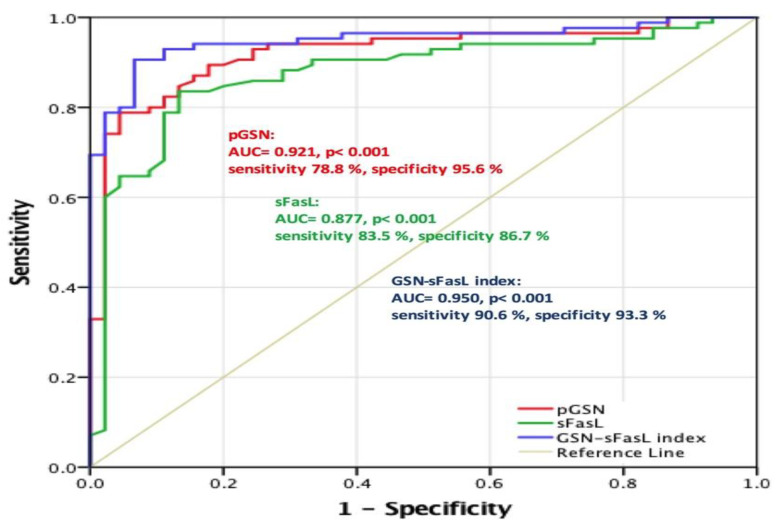
Receiver operating characteristic (ROC) curves of various candidate biomarkers in early HNC prediction. In 85 patients with HNC and 45 healthy controls, with optimal cutoff levels set at 106.13 μg/mL (determined using Fisher’s exact test), circulating pGSN demonstrated a 78.8% sensitivity and 95.6% specificity in early HNC prediction, whereas with optimal cutoff at 30.15 pg/mL, circulating sFasL exhibited an 83.5% sensitivity and 86.7% specificity in early HNC prediction. When pGSN and sFasL were combined to serve as an integrated predictive biomarker set (pGSN–sFasL index) for early HNC stage, with optimal cutoff levels set at 3614.07, the sensitivity and specificity were 90.6% and 93.3%, respectively. Moreover, in the multivariate index assay, pGSN–sFasL index could aid in discriminating patients at early HNC stages more effectively than in discriminating controls, with AUC >0.9 (*p* < 0.001).

As circulating pGSN levels were much lower in patients with early-stage HNC than in healthy controls, we further tested how reliably pGSN could be used as an early diagnostic biomarker for HNC. Here, the approximate AUC derived from the ROC curve was used to assess the diagnostic performance of pGSN (Figure 3). In addition, ROC curve analyses for combined biomarkers (the purple line) were compared. On the basis of the 85 patients with early-stage HNC and 45 healthy controls, the AUC of pGSN for predicting early HNC stage was 0.921 (*p <* 0.001), whereas that of sFasL was 0.877 (*p* < 0.001). With the cutoff level set at 106.1 μg/mL (i.e., the optimal cutoff level derived from Fisher’s exact test), circulating pGSN demonstrated sensitivity and specificity of 78.8% and 95.6%, respectively, for early HNC prediction. They were 83.5% and 86.7%, respectively, using sFasL as a predictor (cutoff level set at 30.2 pg/mL). To further explore whether combination of multiple candidate biomarkers could increase diagnostic accuracy for early HNC stage, levels of circulating pGSN and sFasL were integrated as multivariate index. The pGSN–sFasL index was derived from circulating pGSN and sFasL levels as follows:

pGSN–sFasL index = pGSN level (in μg/mL) × sFasL level (in pg/mL)

The results indicated that the pGSN–sFasL index yielded the highest AUC value (0.950), with a 90.6% sensitivity and 93.3% specificity. Table 1 illustrates the sensitivity and specificity for the discrimination between patients with early-stage HNC and the healthy controls. Taken together, these results demonstrated that circulating pGSN may serve as a diagnostic biomarker for early HNC stages. Furthermore, it presented the optimal sensitivity and specificity in combination with circulating sFasL levels.

### 2.4. Survival Analysis of Circulating pGSN in Validation Datasets

**Figure 4:**
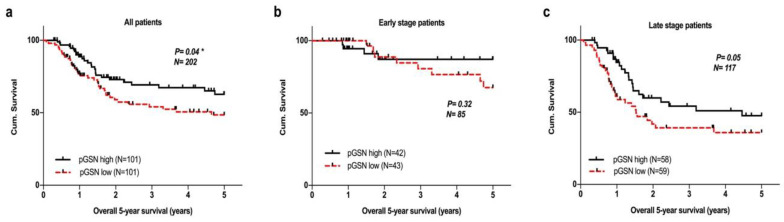
Influence of circulating pGSN levels on 5-year survival of patients with HNC. (**a**) Circulating pGSN in the whole HNC population (*n* = 202) exhibited significant predictive value for 5-year survival, as evaluated using Kaplan–Meier analysis (*p* = 0.04). Higher circulating pGSN levels predicted superior clinical outcomes than did lower pGSN levels. (**b**) Circulating pGSN levels had no significant effect on the overall survival (OS) of early-stage patients with HNC. (**c**) High circulating pGSN expression tends to confer longer OS in late-stage subgroup than those with lower pGSN levels (*p* = 0.05). The *p* values were calculated using the log-rank test.

We further explored the predictive value of circulating pGSN in the survival of patients with HNC. Circulating pGSN levels were dichotomized using its medians (73.5 µg/mL) into low- and high-level groups. The Kaplan–Meier survival analysis results indicated that patients with high pGSN levels (*n* = 101) had significantly higher 5-year OS than did those with lower pGSN levels (*n* = 101; *p* = 0.04; Figure 4a). However, no significant difference was noted in the 5-year survival of early-stage patients with HNC (Figure 4b). Higher pGSN levels had a positive impact on the 5-year OS in late-stage subgroup compared with lower pGSN levels; this result was nearly reached statistical significance (*p* = 0.05; Figure 4c).

**Figure 5:**
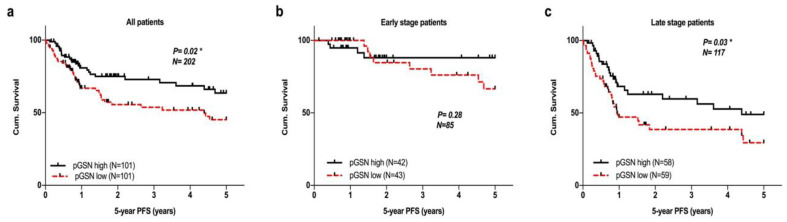
Five-year progression-free survival (PFS) of patients with HNC stratified by the median of circulating pGSN. (**a**) Kaplan–Meier survival analysis stratified by circulating levels of pGSN in the entire HNC population (*n* = 202) revealed that patients with higher circulating pGSN levels demonstrated enhanced 5-year PFS than did those with lower pGSN levels (*p* = 0.02). (**b**) pGSN levels failed to present a significant influence on 5-year PFS of early-stage patients. (**c**) For patients at late HNC stage (*n* = 117), pGSN presented significant predictive value for the 5-year PFS in patients with HNC (*p* = 0.03). The *p* values were calculated using the log-rank test.

Recurrence in advanced HNC is not uncommon. When we further assess patients’ 5-year progression-free survival, we observed the significant predictive value of pGSN for their 5-year progression-free survival (PFS) (*p* = 0.02; Figure 5a). Furthermore, higher circulating pGSN levels predicted superior clinical outcomes than did lower pGSN levels. Circulating pGSN levels did not present a significant effect on 5-year PFS for patients at early HNC stage (*n* = 85; Figure 5b). For patients at late HNC stage (*n* = 117), pGSN presented significant predictive value for the 5-year PFS (*p* = 0.03, Figure 5c).

### 2.5. Prognostic Impact of Circulating pGSN and Its Relationship with Other Clinicopathological Parameters

We further evaluated the prognostic impact of pGSN and other clinicopathologic parameters by using univariate and multivariate Cox regression analyses, as shown in Supplementary Table S3. In the univariate analysis, TNM stage, TNM_T, TNM_N, and pGSN level were significantly associated with PFS and OS. In the multivariate Cox regression analysis, pGSN level (hazard ratios (HR), 1.78; 95% confidence interval (CI), 1.07–2.95; *p* = 0.025) and TNM_N (HR, 2.81; 95% CI, 1.49–5.28; *p* = 0.001) were significant predictors of PFS. Similarly, pGSN level (HR, 1.87; 95% CI, 1.08–3.25; *p* = 0.026) and TNM_N (HR, 2.50; 95% CI, 1.25–4.99; *p* = 0.01) were significantly associated with an increased mortality risk of death.

## 3. Discussion

Mortality in HNC increases considerably when the disease is diagnosed in its late stages. Therefore, early detection of the malignancy is critical to prolonging the OS of patients. However, no reliable biomarkers currently exist for early HNC detection, and the absence of clinical symptoms or signs at early disease stages poses great challenges in HNC screening programs. It is thus conceivable that the discovery of more sensitive and specific HNC biomarkers at early disease stages would be of great value. These biomarkers may not only assist in timely clinical interventions to achieve favorable clinical outcomes but also provide quality of life of the patients that otherwise would suffer from significant complications of cancer treatment.

On the basis of their impact, cancer biomarkers can be divided into diagnostic, predictive, prognostic, and therapeutic biomarkers [26]. Several cellular or molecular biomarkers have been reported for HNC detection with varying degrees of specificity and sensitivity, including p16, matrix metalloproteinase 9, and human papillomavirus [5,6,7]. However, none of these biomarkers have been practically used in the clinical settings because of a lack of adequate clinical relevance to therapeutic implications for early diagnosis. Although SCC-Ag is a potential serum prognostic biomarker for HNC, its detection sensitivity and specificity are insufficient for serving as a diagnostic biomarker [27,28].

GSN is an evolutionary highly conserved protein in vertebrates and exists in four isoforms. cGSN has multifunctional roles in various physiological and pathological processes such as regulation of actin cytoskeleton dynamics, cell motility, and metastasis [29,30]. Furthermore, cGSN plays critical roles in apoptosis signaling and cell differentiation [31]. In large-scale clinical studies, Shieh et al. reported that tissue cGSN expression is an independent prognostic biomarker in stage-I non-small-cell lung [32] and oral [33] cancers. The prognostic value of cGSN has further been confirmed in several other cancers, including colon, ovarian, and breast cancers [22,23,34]. We recently discovered that cGSN expression is highly associated with chemoresistance phenotype in both HNC and ovarian cancers by suppressing apoptosis induced by chemotherapeutic agents [15,16].

On the other hand, pGSN plays a critical role in preventing the polymerization of actin released from damaged tissues and thereby trigger platelet aggregation and microvascular thrombosis [35,36]. Notably, clinical studies have indicated lower circulating pGSN levels in patients with trauma, acute respiratory distress syndrome, sepsis, and acute liver failure [19,20,21]. The prognostic value of circulating pGSN was later confirmed under various clinical conditions. Horváth-Szalai et al. [37] reported that pGSN and actin/GSN ratio may represent an efficient complementary prognostic marker for sepsis. Asare-Werehene and colleagues [38] recently demonstrated that exosomal pGSN could promote the survival of ovarian cancer cells through both autocrine and paracrine stimulation and thereby confer chemoresistance. Moreover, preoperative circulating pGSN was found to be a favorable biomarker for early ovarian cancer detection, residual disease prediction, and overall prognosis [39]. Chen et al. [40] also reported that serum pGSN levels were significantly lower in patients with colon cancer than in healthy controls and that pGSN could serve as a more effective diagnostic biomarker for colon cancer compared with the currently used carcinoembryonic antigen. However, the role of pGSN in HNC has not been elucidated.

In the present study, we found that circulating pGSN can serve as a novel diagnostic biomarker for the detection of HNC. Compared with other reported potential HNC biomarkers, pGSN had far superior sensitivity (82.7%) and specificity (95.6%) for HNC diagnosis. In addition, the level of sFasL, a vital apoptosis signaling molecule, was significantly lower in patients with HNC. It presented the optimal detection sensitivity (83.2%) and the second highest specificity (86.9%) for HNC. This discovery is consistent with previous studies that reported lower sFasL levels in patients with HNC [41].

Furthermore, we assessed the diagnostic values of pGSN and sFasL for early HNC detection. Circulating pGSN presented higher specificity (95.6%) than did sFasL levels (86.7%) for early HNC stage, whereas sFasL presented higher sensitivity (83.5%) than did pGSN (78.8%). We combined pGSN and sFasL levels to form a new index (pGSN–sFasL index), which provided augmented sensitivity (90.6%) compared with each individual biomarker for detecting early-stage HNC. Yu et al. reported the use of a four-protein panel (MMP1, KNG1, ANXA2, and HSPA5) to evaluate cancer progression risk and early disease detection of oral squamous cell carcinoma (OSCC) using liquid chromatography–multiple-reaction-monitoring–mass spectrometry. The panel achieved a detection sensitivity of 88.6% and specificity of 80.4% for early-stage OSCC [42]. Our results indicated that circulating pGSN could serve as an independent biomarker for early HNC detection with superior specificity compared with the four-protein panel of Yu et al.; furthermore, much greater sensitivity (90.6%) and specificity (93.3%) were obtained after using the pGSN–sFasL index. Furthermore, circulating pGSN levels also provide promising value in clinical outcome prediction, and thus, it may serve to guide clinical disease management. We demonstrated that higher pGSN levels predict significantly favorable survival than do lower pGSN levels in both the 5-year OS and PFS follow-up analyses.

The mechanisms underlying the considerable value of pGSN in the early detection and prediction of clinical outcomes related to malignant diseases may be derived from its critical functions in various carcinogenesis-associated pathways. Numerous studies have reported crosstalk between tissue inflammation and cancer initiation and progression. Fas ligand/receptor signaling plays critical roles in the regulation of the immune system and cancer progression [43]. Consistent with our findings, Hoffmann et al. reported lower sFasL levels in patients with HNC and suggested that serum sFasL is consumed by binding to Fas expressed on activated circulating CD8+ T lymphocytes in patients with cancer, thereby reducing their sFasL levels compared to healthy controls [44]. Clinical studies have reported a strong association between circulating pGSN levels and inflammation-associated clinical states, but their potential regulatory roles in relation to immune cells were only revealed recently. pGSN was reported to inactivate CD4+ T cells through CD37 signaling and to induce the apoptosis of activated CD8+ T cells through membrane-bound FasL [24]. Similarly, the observed lower circulating pGSN level in patients with HNC compared with in healthy controls could reflect their consumption by CD8+ T cells that triggered apoptosis. This explained a potential mechanism that a higher pGSN level predicted a more favorable prognosis.

Despite satisfactory advancements in sensitivity and specificity from using pGSN as a circulating biomarker, as presented in our study, saliva should be used instead of blood for cancer screening for better compliance and straightforward collection and transportation. The existence of salivary GSN has been reported by Bermejo-Pareja et al. [45]. However, its clinical relevance related to HNC has not been investigated. Further investigation is warranted.

Although chronic inflammation is a risk factor for malignancy, including HNC [46,47], this is the first report in which circulating pGSN conferred excellent diagnostic value for early-stage HNC, particularly in combination with circulating sFasL. Furthermore, this is the first study to report the prognostic significance of circulating pGSN for HNC after the discovery of cGSN expression in tumor tissues as a prognostic indicator in HNC. The association between pGSN and cGSN and the underlying mechanism are anticipated to have significant effects on HNC clinical disease management and remain to be explored further.

## 4. Materials and Methods

### 4.1. Ethics Statement

The current clinical study protocol and consent forms were approved by the Institutional Review Board of National Cheng Kung University Hospital, Taiwan (IRB approval number: A-ER-106-505) and the Human Research Ethics Committee of National Cheng Kung University. The experiment was conducted in accordance with relevant guidelines and the need for informed consent for the collection, analysis, and publication of patient data was waived.

### 4.2. Clinicopathological Characteristics and Collection of Plasma Samples

We included 202 patients who were diagnosed as having HNC between April 1998 and January 2015 at National Cheng Kung University Hospital, Taiwan. These patients underwent comprehensive staging or cytoreductive surgery with adjuvant chemotherapy. Staging was performed using the TNM staging system according to the American Joint Committee on Cancer, eighth edition classification. Cancer progression was defined according to the objective Response Evaluation Criteria in Solid Tumors (RECIST version 1.1). Two pathologists independently reviewed the medical records and pathological slides, which provided information on patent demographics, clinical characteristics, pathological diagnoses, and treatment outcomes. Control blood samples were obtained from 45 healthy individuals who were confirmed to be without cancer, suspected cancer, or inflammatory conditions and agreed to participate in the study. Before neoadjuvant chemotherapy or radiotherapy, all the plasma samples were collected in blood collection tubes containing EDTA as an anticoagulant and then centrifuged for 15 min at 1000× *g*. The samples were separated and placed in 1.5-mL Eppendorf aliquots and stored at −80 °C, and the plasma levels of all the included cancer biomarkers were measured.

Survivor data were censored on the date on which the survivors were last known to be alive. The OS was calculated from the date of diagnosis to the date of death from any cause. The PFS was calculated from the first treatment of HNC to the date of disease progression or death from any cause, unless the patient was progression free at the time of last contact; in that case, the progression-free interval was measured to the date of last contact. The participants were followed up after treatment, and the date of the latest record retrieval was May 31, 2019. OS and PFS were stratified by circulating pGSN levels during follow-up.

### 4.3. Circulating pGSN and SCC-Ag Detection from Patient Plasma Using Sandwich ELISA

pGSN and SCC-Ag levels in both patients with HNC and healthy controls were determined using a Plasma Gelsolin ELISA Kit (SK00384-06; Aviscera Bioscience, CA, USA) and a Human Squamous Cell Carcinoma Antigen ELISA Kit (MBS-162089; MyBioSource, SD, USA), respectively, according to manufacturer instructions. Briefly, all plasma samples were diluted in a sample buffer (5000× for pGSN, Aviscera Bioscience; 5× for SCC-Ag, MyBioSource) and placed in an ELISA plate pre-coated with a capture antibody specific for human target proteins pGSN or SCC-Ag. The samples were washed four times using wash buffer. The presence of target proteins in the samples were then probed using biotinylated monoclonal antibodies against target proteins specifically. After the plate was further washed again, HRP-conjugated streptavidin was added; this was followed by a last wash to remove any unbound enzyme. The plate was developed by adding substrate solution (TMB) for color development and quantified with reference to the standard curve of optical density at 450 nm in a microplate reader. A standard curve was created using computer software, which could generate a four-parameter logistic curve fit. Each specimen was converted to pGSN or SCC-Ag levels using this standard curve.

### 4.4. Circulating Biomarker Detection

Expression of circulating cancer biomarkers was assessed, as described previously [48]. The levels of three proteins (CYFRA21-1, sFas, and sFasL) were determined using the cancer biomarker panel according to the protocol provided by the manufacturer (Milliplex Map Human Circulating Cancer Biomarker Magnetic Bead Panel HCCBP1MAG-58K; Millipore, St. Charles, MO, USA). Plasma samples were diluted to double the volume using the serum matrix provided in the kit as a sample diluent. In brief, the microplate was primed using assay buffer and a diluted sample or control plasma was added; subsequently, it was mixed well with the magnetic bead and incubated with agitation overnight at 4 °C. The plate was gently washed before the detection antibodies were added and then incubated for 1 h at room temperature. Next, streptavidin-phycoerythrin was added to each well before the plate was incubated for 30 min and washed three times. Sheath fluid was added to all wells and then the plate was analyzed in the multiplexing instrument Luminex^®^200™ (Invirtogen, Merelbeke, Belgium) using the software package xPONENT (Luminex Corporation, Austin, TX, USA). The median fluorescent intensity was obtained using a five-parameter logistic function for calculating the analyte levels in the samples.

### 4.5. Data Analysis

All statistical analyses were performed using SPSS (version 24.0; SPSS Inc., Chicago, IL, USA) and Prism (version 7; GraphPad, San Diego, CA, USA). Mean pGSN and other biomarkers levels were plotted against their respective disease states using box plots. The statistical analysis was performed using unpaired *t*-tests and one-way analysis of variance. ROC curves were used to assess the discriminating capacity of pGSN and other cancer biomarkers. AUC was considered the capacity for disease classification from healthy controls. OS and PFS curves were plotted with Kaplan–Meier plots, and *p* values were calculated using the log-rank test. A two-sided *p* ≤ 0.05 was considered to represent statistical significance. Univariate and multivariate Cox proportional hazards models were used to estimate HR and corresponding 95% CI. The independent effect of circulating pGSN levels during follow-up on survival and disease progression was also analyzed. *p* values of < 0.05 (two-sided) were considered significant.

We estimated the sample size required to compare the high and low pGSN groups for death in the Cox regression model. We set type I error α to be 0.05 and power to be 0.8 and then used the event rates (0.3 and 0.4) of two groups and postulated hazard ratio to estimate the sample size. Thus, a sample size of 100 participants for the high pGSN group and 100 for the low pGSN group had reasonable statistical power (0.8) in our study. We conducted Cox proportional hazard models to control for potential confounding factors (sex, age, differentiation, tumor size, TNM_T, TNM_N, and staging) to assess the HRs (with corresponding 95% CIs) between high and low pGSN groups. A multivariate Cox regression analysis including all variables achieved significant results (*p* < 0.05) in the univariate analysis. The proportional hazard assumption was tested using Schoenfeld residuals, and it was valid for all outcomes [49].

## 5. Conclusions

We discovered that circulating pGSN levels in HNC patients were lower than those in normal healthy controls. Circulating pGSN could serve as a diagnostic biomarker for HNC, especially for early disease stages, with augmented sensitivity and specificity in combination with circulating sFasL. Furthermore, lower circulating pGSN levels predicted significantly poorer clinical outcomes (i.e., shorter 5-year OS and PFS). Taken together, circulating pGSN is a potential independent predictor of favorable clinical outcomes and a novel biomarker for the early HNC detection in combination with sFasL.

## Figures and Tables

**Table 1:** Sensitivity and specificity of pGSN, sFasL, or their combination in discriminating between early HNC stage and healthy groups.

Variables	AUC	Significance	Sensitivity (%)	Specificity (%)
All patients				
pGSN	0.937	< 0.001	82.7	95.6
Early-stage patients				
pGSN	0.921	< 0.001	78.8	95.6
sFasL	0.877	< 0.001	83.5	86.7
pGSN–sFasL index	0.950	< 0.001	90.6	93.3

## Supplementary Materials

The following are available online at https://www.mdpi.com/2072-6694/12/6/1569/s1, Table S1: Patient Characteristics for HNC, Table S2: Tumor sites of the HNC patients, Table S3: Crude and adjusted HR for OS and PFS.
