# Supplementary material for: Circulating Plasma Gelsolin: A Predictor of Favorable Clinical Outcomes in Head and Neck Cancer and Sensitive Biomarker for Early Disease Diagnosis Combined with Soluble Fas Ligand
Source: Cancers (Basel). 2020 Jun 13;12(6):1569. doi: 10.3390/cancers12061569 (PMC7353036; doi:10.3390/cancers12061569)
Supplement: Supplementary file 1 [file cancers-12-01569-s001.pdf]

cancers

Circulating Plasma Gelsolin: A Predictor of Favorable Clinical Outcomes in Head and Neck Cancer and Sensitive Biomarker for Early Disease Diagnosis Combined with Soluble Fas Ligand

Chen-Tzu Chiu [1,†], Pei-Wen Wang [2,3,†], Meshach Asare-Werehene [4,5], Benjamin K. Tsang [4,5] and Dar-Bin Shieh [1,2,3,6,*]

1 Institute of Basic Medical Sciences, National Cheng Kung University, Tainan 70101, Taiwan; dinosaurchiu@gmail.com
2 Institute of Oral Medicine and Department of Stomatology, College of Medicine, National Cheng Kung University Hospital, National Cheng Kung University, Tainan 70101, Taiwan; peiwen1005@gmail.com.tw
3 Center of Applied Nanomedicine, National Cheng Kung University, Tainan 70101, Taiwan
4 Departments of Obstetrics & Gynecology and Cellular & Molecular Medicine, University of Ottawa, Ottawa, ON, Canada; masarewerehene@yahoo.com(M.A-W.), btsang@ohri.ca (B.K.T.)
5 Chronic Disease Program, Ottawa Hospital Research Institute, Ottawa, ON K1H 8L6, Canada
6 Center for Micro/Nano Science and Technology, Advanced Optoelectronic Technology Center, Innovation Center for Advanced Medical Device Technology, National Cheng Kung University, Tainan 70101, Taiwan

* Correspondence: darbinshieh@gmail.com; Tel.: +886-6-235-3535 (ext. 5899)
† Equal contribution

Received: 11 May 2020; Accepted: 09 June 2020; Published: date

Table S1. Patient Characteristics for HNC.

Characteristics	Number of Patients (%)
Age (Range; 22–81 years)	
≤ 53	105 (52%)
> 53	97 (48%)
Differentiation	
Well	104 (51.5%)
Moderate	73 (36.1%)
Poor	17 (8.4%)
Not verified	8 (4%)
TNM stage	
I	33 (16.3%)
II	52 (25.7%)
III	40 (19.8%)
IV	77 (38.1%)
TNM stage	
≤ 2	85 (42.1%)
> 2	117 (58%)
Recurrence	
No	147 (72.8%)
Yes	55 (27.2%)
Vital status	
Alive	126 (62.4%)
Dead	76 (37.6%)

Table S2. Tumor sites of the HNC patients.

**Tumor site**	**Total (%)**	**TNM Stage**	
		**Early (%)**	**Late (%)**
Buccal	78 (38.6%)	35 (44.6%)	43 (55.4%)
Tongue	72 (35.6%)	28 (37.8%)	44 (62.3%)
Gingivae	30 (14.8%)	12 (41.4%)	18 (58.6%)
Lip	8 (4%)	3 (37.5%)	5 (62.5%)
Maxilla	4 (2%)		
Floor of mouth	3 (1.5%)		
Retro-molar	1 (0.5%)		
Others	6 (3%)		

Table S3. Crude and adjusted HR for OS and PFS.

	**Low pGSN**	**High pGSN**	***p* value**
	***n* = 101**	***n* = 101**	
**Event rate**	39 (38.61%)	26 (25.74%)	
**OS**			
Crude HR (95% CI)	1.68 (1.02–2.76)*	1	0.041
Adjusted HR (95% CI)	1.87 (1.08–3.25)*	1	0.026
**PFS**			
Crude HR (95% CI)	1.78 (1.09–2.92)*	1	0.021
Adjusted HR (95% CI)	1.78 (1.07–2.95)*	1	0.025

HR, hazard ratio; CI, confidence interval; OS, overall survival; PFS, progression-free survival. *Estimated from Cox proportional hazard regression model. Significant variables ($p < 0.05$) in univariate Cox regression model were included in the multivariate analysis. Multivariable Cox proportional hazard model was adjusted for TNM_T, TNM_N and stage.
